# Pulsed-Field Ablation of Confluent Inferior Pulmonary Vein With Narrowing and a Low-Voltage Area: A Case Report

**DOI:** 10.7759/cureus.108012

**Published:** 2026-04-30

**Authors:** Yuki Tanaka, Masaru Yamaki, Yoshifumi Mizuguchi, Yasumi Igarashi

**Affiliations:** 1 Cardiology, Sapporo-Kosei General Hospital, Sapporo, JPN

**Keywords:** atrial fibrillation, inferior common trunk, prevention, pulmonary vein isolation, pulsed-field ablation

## Abstract

Confluent inferior pulmonary vein (CIPV) often has narrow ostia and low-voltage areas in the common trunk. We treated a man in his 60s with atrial fibrillation. Cardiac computed tomography revealed a CIPV with a 5 mm narrow left inferior pulmonary vein (PV) ostium. Electroanatomical mapping showed low-voltage areas in the common trunk. Using guidewire-assisted PulseSelect™ pulsed-field ablation (PFA), we performed PV isolation and left atrial posterior wall isolation. CIPV often manifests as narrow left inferior PV ostia, thin posterior walls, and low-voltage or scar areas in the common trunk. PFA, particularly with the PulseSelect™ system, is well-suited to minimizing PV stenosis and the risk of esophageal injury.

## Introduction

Pulsed-field ablation (PFA) procedures, compared with conventional thermal ablation, are generally shorter in duration and carry a lower risk of collateral thermal injury to the esophagus or phrenic nerve [1,2]. In terms of safety, PFA has never been associated with esophageal events or pulmonary vein (PV) stenosis [3]. Confluent inferior pulmonary vein (CIPV) is a rare anatomic variant that may present technical challenges during PV isolation due to narrow ostia and proximity to the esophagus [4,5]. We report a case of successful guidewire-assisted PFA in a patient with CIPV, with significant ostial narrowing and low-voltage areas in the common trunk.

## Case presentation

A male patient in his 60s with a history of myocardial infarction, hypertension, and dyslipidemia developed exertional palpitations lasting >3 months. His body mass index was 21.2 kg/m², and his CHADS2 and CHADS2-VASC scores were 2 and 3 (congestive heart failure, hypertension, and vascular disease), respectively.

**Figure 1:**
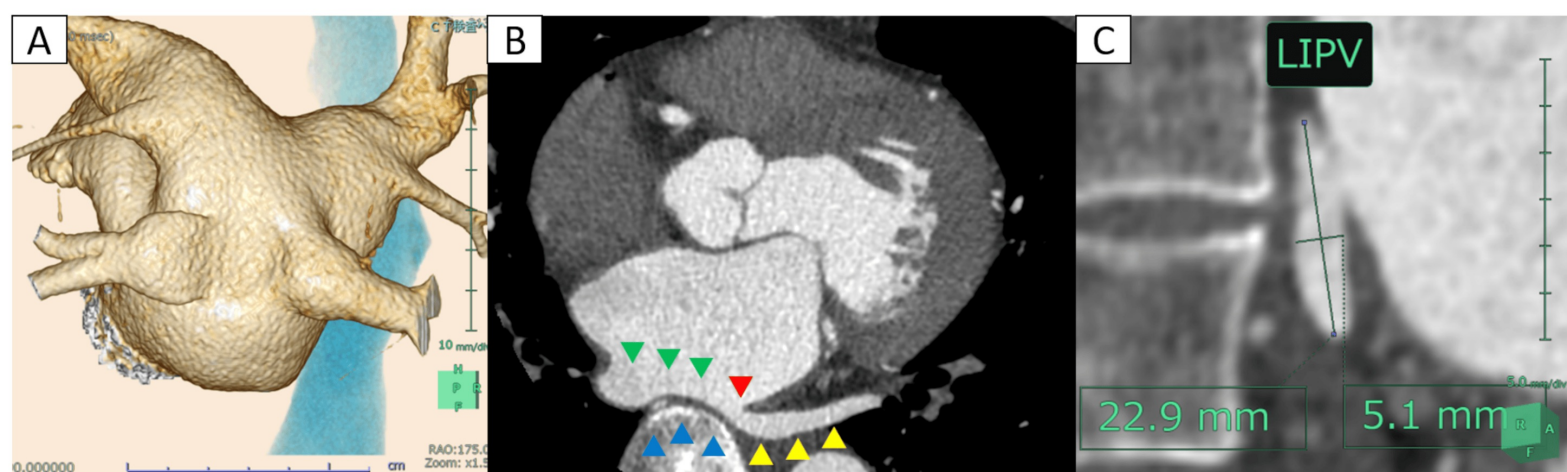
Contrast cardiac CT image. (A) Contrast cardiac CT image of the PA view showing the left atrium and PVs. (B) Contrast cardiac CT image of the axial slice. The blue arrowheads indicate the rim of the spine; the green arrowheads show the common trunk of both inferior PVs; the yellow arrowheads show the left inferior PV; and the red arrowhead shows the narrow ostium of the left inferior PV. The left inferior PV and the common trunk are compressed by the spine. (C) Contrast cardiac CT image of the axial slice to the left inferior PV ostium. The long and short diameters of the left inferior PV ostium measured 22.9 and 5.1 mm, respectively. CT = computed tomography; PA = posterior-anterior; PV = pulmonary vein; LIPV = left inferior pulmonary vein

Electrocardiography (ECG) showed atrial fibrillation (AF), after which the patient’s B-type natriuretic peptide (BNP) level increased from 69.9 to 418.6 pg/mL. He was given amiodarone and edoxaban, which slightly improved his symptoms as his heart rate was controlled. However, this did not address all his symptoms, and he remained at European Heart Rhythm Association score 2b, as his AF persisted three months after the prescription. We, therefore, decided to attempt catheter ablation. Chest radiography revealed a cardiothoracic ratio of 55%. Echocardiography revealed a left ventricular ejection fraction of 36% with akinesis of the anteroseptal wall motion, a left atrial diameter of 44 mm, and mild mitral regurgitation. Contrast-enhanced cardiac computed tomography (CT) revealed that both inferior PVs originated from the common trunk (Figure 1A). The common trunk and ostium of the left inferior PV were compressed by the spine (Figure 1B). The diameter of the left inferior PV ostium was 22.9 × 5.1 mm (Figure 1C).

**Figure 2:**
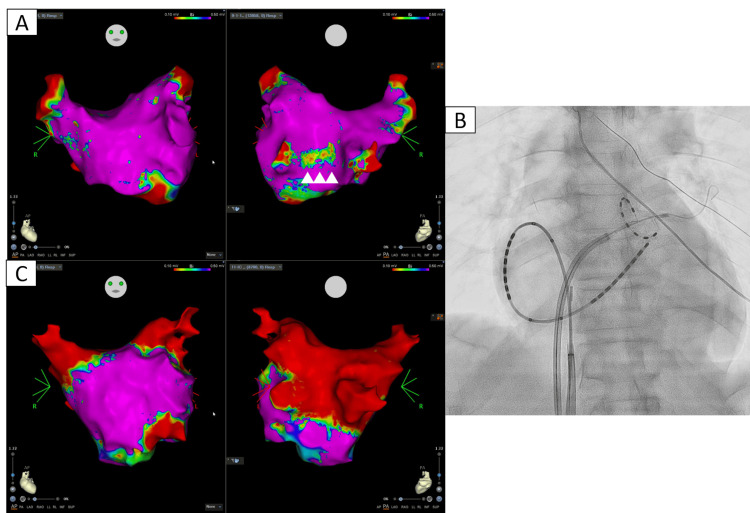
Voltage mapping and fluoroscopic image of the PFA. (A) Pre-voltage mapping using the CARTO 3D electroanatomical mapping system. Left, AP view; right, PA view. Voltage mapping demonstrated that the common trunk of both inferior PVs comprised a low-voltage area (white arrowheads). (B) Fluoroscopic image of the PFA to left inferior PV using the PulseSelect™. (C) Post-voltage mapping of the CARTO 3D electroanatomical mapping system. Left, AP, and right, PA view. Isolation was performed of the PV and left atrial posterior wall, including the common trunk. 3D = three-dimensional; AP = anterior-posterior; PA = posterior-anterior; PV = pulmonary vein; PFA = pulsed-field ablation

**Figure 3:**
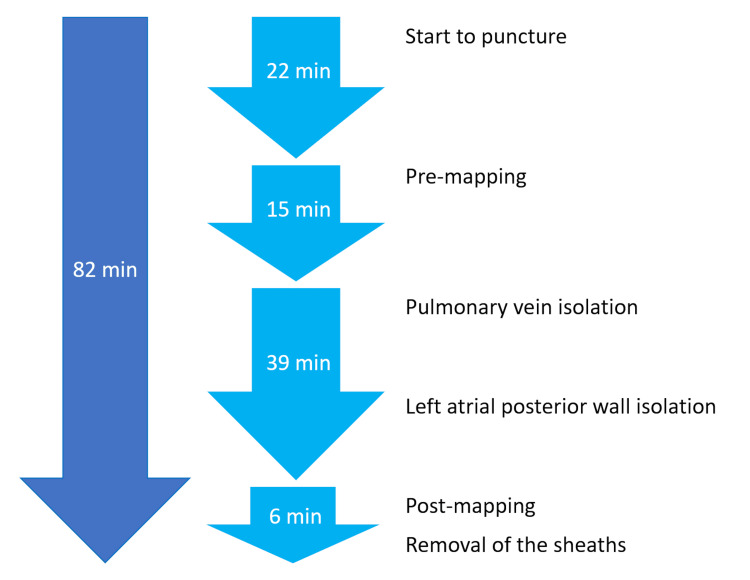
Timeline of the pulsed-field ablation procedure. The total procedure time was 82 minutes.

After obtaining written informed consent from the patient, PFA was performed using a three-dimensional electro-anatomical mapping system (CARTO; Biosense Webster, Diamond Bar, CA, USA). A 20-electrode atrial cardioversion catheter (BeeAT; Japan Lifeline Co. Ltd., Saitama, Japan) was inserted through the right femoral vein. Following the standard Brockenbrough technique, electro-anatomical mapping was performed using an 8-spline high-density mapping catheter (Octaray; Biosense Webster). Low-voltage and scar areas were defined as those with amplitudes of 0.1-0.5 mV and <0.1 mV, respectively. Voltage mapping performed during atrial pacing from the high right atrium demonstrated that the common trunk of these PVs was a low-voltage area (Figure 2A). We thought this low-voltage area could be the isthmus of atrial tachycardia after PV isolation and could be considered for left atrial posterior wall isolation (LAPWI). We, therefore, elected to use a PFA system to avoid PV occlusion of the left inferior PV and performed LAPWI to prevent any esophageal events. Ablation of the left inferior narrow PV was easily achieved using the guidewire-assisted PulseSelect™ PFA system (Medtronic, Minneapolis, MN, USA). We adjusted the co-axiality of a round catheter to fit the left inferior narrow PV ostium shown in the three-dimensional mapping system. This allowed the guidewire to be easily advanced into the PV. We were then able to advance the PulseSelect™ catheter, with a slight alteration to a linear shape, and apply the pulse-field without overlapping the electrodes in the narrow PV ostium (Figure 2B). After performing ablation of the four PVs, we proceeded to conduct LAPWI as well. Post-voltage mapping showed no potential in the four PVs or left atrial posterior wall, including the common trunk of both inferior PVs (Figure 2C). The timeline of the PFA procedure is shown in Figure 3.

The patient was discharged from our institution two days after the procedure, without any complications. There was no atrial tachycardia recurrence on 12-lead follow-up ECGs or development of any palpitations at one, three, and six months after the procedure. Moreover, his BNP level decreased to 40.1 pg/mL, and cardiothoracic ratio on chest radiography improved to 50% at six months’ follow-up. These findings also support the effectiveness of catheter ablation.

## Discussion

The non-inferiority of the acute and mid-term clinical outcomes of PFA versus thermal ablation has been previously confirmed [6,7]. PFA is also a safe approach that avoids complications such as esophageal events, PV stenosis, or phrenic paralysis [3]. Therefore, we propose that clinicians consider PFA for index catheter ablation procedures to treat AF with PV narrowing or planned LAPWI.

CIPV is a rare variation detectable by CT in 0.7-1.5% of patients with AF who undergo PV isolation [4,8]. In the present case, the short axial diameter of the left inferior PV was only 5.1 mm as a result of compression by the spine. The ostium of the left inferior PV can sometimes be compressed by the esophagus or aorta in cases of CIPV [5]. PFA treatment is, therefore, suitable for ablation in such cases as well. We elected a guidewire-assisted PFA approach, which would facilitate the selection of a narrow PV ostium when PV ostial ablation was performed. When we adjusted the coaxiality of a round catheter to the PV ostium observed on the three-dimensional mapping system, we were easily able to advance the guidewire into the narrow PV. Moreover, we were able to advance the PulseSelect™ catheter with a slight alteration to a linear shape in the narrow PV. This system also allows pulsed fields to be applied, without overlapping the electrodes used, within narrower PV ostia. Therefore, we thought the PulseSelect™ system was favorable for guidewire-assisted PFA for narrow PV ostia.

Patients with CIPV have a thinner posterior wall thickness than normal, suggesting a high risk of esophageal injury [4]. Moreover, CIPV may not show detectable potential in the inferior PVs or inferior common trunk [9]. This finding could become the isthmus of certain atrial tachycardias, such as the roof-dependent one sometimes observed following PV isolation [10]. Therefore, LAPWI should be considered for thin left atrial posterior walls in certain CIPV cases. Indeed, CIPV is often treated via circumferential isolation, similarly to the LAPWI approach [4,5,8,9]. In these aspects, we determined that PFA was favorable for catheter ablation to treat the present case of AF with CIPV. For these reasons, we thought PulseSelect™ could be a suitable device for performing PFA in cases of CIPV.

## Conclusions

We recently used PFA, using the PulseSelect™ system, to successfully treat a case of CIPV with left inferior PV narrowing and a low-voltage area in the common trunk. CIPV often involves narrowing of the left inferior PV, a thin left posterior wall, or areas of low voltage or scars in the common trunk. PFA, particularly using the PulseSelect™ system, is well-suited to addressing these CIPV-related challenges.

## References

[1] de Campos MC, Moraes VR, Daher RF (2024). Pulsed-field ablation versus thermal ablation for atrial fibrillation: a meta-analysis. Heart Rhythm O2.

[2] Amin AM, Nazir A, Abuelazm MT (2024). Efficacy and safety of pulsed-field versus conventional thermal ablation for atrial fibrillation: a systematic review and meta-analysis. J Arrhythm.

[3] Ekanem E, Neuzil P, Reichlin T (2024). Safety of pulsed field ablation in more than 17,000 patients with atrial fibrillation in the MANIFEST-17K study. Nat Med.

[4] Negishi K, Okumura K, Onishi F (2024). Posterior wall thickness of the confluent inferior pulmonary veins measured by left atrial intracardiac echocardiography: implications for catheter ablation. J Interv Card Electrophysiol.

[5] Yu R, Dong J, Zhang Z (2008). Characteristics in image integration system guiding catheter ablation of atrial fibrillation with a common ostium of inferior pulmonary veins. Pacing Clin Electrophysiol.

[6] Reddy VY, Gerstenfeld EP, Natale A (2023). Pulsed field or conventional thermal ablation for paroxysmal atrial fibrillation. N Engl J Med.

[7] Reichlin T, Kueffer T, Badertscher P (2025). Pulsed field or cryoballoon ablation for paroxysmal atrial fibrillation. N Engl J Med.

[8] Yamane T, Date T, Tokuda M (2008). Prevalence, morphological and electrophysiological characteristics of confluent inferior pulmonary veins in patients with atrial fibrillation. Circ J.

[9] Kokubu Y, Watanabe T, Yamada T (2024). A rare case of a common inferior pulmonary vein presumed to be a remnant of the common pulmonary vein. Intern Med.

[10] Tohoku S, Chun KR, Bordignon S (2023). Findings from repeat ablation using high-density mapping after pulmonary vein isolation with pulsed field ablation. Europace.

